# Fundamentals of FGF19 & FGF21 Action *In Vitro* and *In Vivo*

**DOI:** 10.1371/journal.pone.0038438

**Published:** 2012-05-31

**Authors:** Andrew C. Adams, Tamer Coskun, Armando R. Irizarry Rovira, Michael A. Schneider, David W. Raches, Radmila Micanovic, Holly A. Bina, James D. Dunbar, Alexei Kharitonenkov

**Affiliations:** Lilly Research Laboratories, Lilly Corporate Center, Indianapolis, Indiana, United States of America; University of Hong Kong, China

## Abstract

Fibroblast growth factors 19 (FGF19) and 21 (FGF21) have emerged as key regulators of energy metabolism. Several studies have been conducted to understand the mechanism of FGF19 and FGF21 action, however, the data presented has often been inconsistent and at times contradictory. Here in a single study we compare the mechanisms mediating FGF19/FGF21 actions, and how similarities/differences in actions at the cellular level between these two factors translate to common/divergent physiological outputs. Firstly, we show that in cell culture FGF19/FGF21 are very similar, however, key differences are still observed differentiating the two. In vitro we found that both FGF's activate FGFRs in the context of βKlotho (KLB) expression. Furthermore, both factors alter ERK phosphorylation and glucose uptake with comparable potency. Combination treatment of cells with both factors did not have additive effects and treatment with a competitive inhibitor, the FGF21 delta N17 mutant, also blocked FGF19's effects, suggestive of a shared receptor activation mechanism. The key differences between FGF21/FGF19 were noted at the receptor interaction level, specifically the unique ability of FGF19 to bind/signal directly via FGFR4. To determine if differential effects on energy homeostasis and hepatic mitogenicity exist we treated DIO and ob/ob mice with FGF19/FGF21. We find comparable efficacy of the two proteins to correct body weight and serum glucose in both DIO and ob/ob mice. Nevertheless, FGF21 and FGF19 had distinctly different effects on proliferation in the liver. Interestingly, *in vivo* blockade of FGF21 signaling in mice using ΔN17 caused profound changes in glycemia indicative of the critical role KLB and FGF21 play in the regulation of glucose homeostasis. Overall, our data demonstrate that while subtle differences exist *in vitro* the metabolic effects *in vivo* of FGF19/FGF21 are indistinguishable, supporting a shared mechanism of action for these two hormones in the regulation of energy balance.

## Introduction

In mammals the fibroblast growth factors (FGFs) play diverse roles in the regulation of many cellular processes ranging from development to survival [1], [2]. The FGF superfamily consists of 22 members of which 3 have recently been classified to form an “endocrine” sub-group [3]. This classification is based on the high degree of structural homology between the members of this sub-family FGF19 (FGF15 in mice), FGF21 and FGF23. These hormone-like FGFs lack a conventional heparin binding domain, which allows them to reach the circulation where they are present in measurable amounts. Instead of heparin, FGF19, FGF21, and FGF23 utilize Klotho co-factor proteins to permit binding to and activation of fibroblast growth factor receptors (FGFRs).

Previous publications have demonstrated that FGF15/19 and FGF21 bind to the βKlotho (KLB) isoform of the Klotho family while FGF23 has distinct affinity for the αKlotho (KL) subtype [4]. There has been some discussion of FGF15/19 binding to KL [5], however, this finding has not been replicated by others [6]. While the tissue distribution of FGFRs is relatively widespread there is a discrete pattern of KLB expression mainly in metabolically active tissues such as the liver, pancreas and white adipose tissue. This distribution suggests that it is the presence of KLB rather than the expression of a particular FGFR which determines the tissue specificity of FGF19 and FGF21 action in vivo [6], [7].

FGF23 plays a well described role in phosphate metabolism and has not previously been shown to affect energy balance [8]. However, a large body of literature is now emerging supporting a role for both FGF19 and FGF21 in the regulation of energy homeostasis [3], [9], [10]. FGF19 and FGF21 have both previously been reported to have significant effects on energy homeostasis in obese animals [10]–[12]. However, to date there has not been a direct comparison of the in vivo and in vitro determinants of their actions on metabolism and the relative magnitude of their physiological effects.

We demonstrate here that on both a molecular and whole organism level there are many similarities in the action of FGF19 and FGF21. While FGF21 showed no direct FGFR binding, FGF19 was able to bind FGFR4 independent of KLB. In functional studies we show in 3T3-L1 fibroblasts expressing KLB, both FGF19 and FGF21 were able to stimulate glucose uptake with similar pharmacodynamic properties. When 3T3-L1 adipocytes were treated with a combination of both FGF19 and FGF21 we saw no additive or synergistic effect. Furthermore, treatment with an inhibitory truncated form of FGF21 (termed ΔN17) [13] blocked increases in phosphrylation of extracellular signal-regulated kinase (pERK) and glucose uptake stimulated by both FGF21 and FGF19. We hypothesize that these effects are likely due to the two factors operating upstream via the same FGFR receptor complex(s) in the context of KLB expression. Nevertheless, we also show that FGF19 is not only able to bind, but also to activate FGFR4 directly as measured by phosphorylation of ERK in both FGFR4 over-expressing 3T3-L1 fibroblasts and FGFR4 expressing L6 myoblasts which lack KLB. As FGF19 has previously been reported to induce mitogenicity, we examined the effects of FGF19 and FGF21 in an in vivo BRDU incorporation assay. FGF19 signficantly increased the number of BRDU-positive hepatocellular nuclei in the liver, however, FGF21 had no effect confirming differences in the proliferative properties of the two factors. To determine if the interchangeability we observed at the cellular level carries over to metabolic physiology we treated diet induced obese mice with either FGF19 or FGF21. In DIO animals both treatments lead to a significant but comparable reduction in body mass. Furthermore, we show that serum glucose is lowered in an equivalent manner by both factors at corresponding doses. In ob/ob mice we see a similar pattern in that both FGF19 and FGF21 reduce body mass accrual during the treatment period to a similar extent with a mildly greater effect seen in the FGF21 treated mice. Serum glucose was significantly lowered following treatment in ob/ob mice treated with either FGF19 or FGF21, however, the magnitude of the reduction was not different between the two factors. Finally, we show that ΔN17, which we and others have previously reported is able interact with KLB but unable to induce FGFR activation due to the lack of N-terminus, acts in an antagonistic manner in mice by blocking FGF21 mediated reductions in serum glucose. We go on to show that in the fasted state treatment with ΔN17 alone leads to elevated serum glucose, suggesting a role for FGF21 and KLB in glucose regulation during the normal fed/fasted transition, and establishing KLB as a key molecule required to propagate FGF21 action at the whole body level. Taken as a whole, our data demonstrate that the metabolic actions of the “endocrine” FGFs likely occur via activation of a similar molecular pathway.

## Methods

### Proteins

For both in vitro and in vivo studies FGF19, FGF21 and ΔN17 were generated as previously described [14].

### Animals

All animals were individually housed in a temperature-controlled (24°C) facility with 12 h/12 h light/dark cycle. Animal protocols in this study were approved by the Eli Lilly and Co. Animal Use and Care Committee (Protocol No. 09012).

### FGF treatment of DIO animals

Male C57Bl/6J mice (n = 6 per group) (Taconic Farms) were maintained on a calorie-rich diet consisting of 40% fat, 39% carbohydrate, and 21% protein caloric content (TD95217; Harlan Teklad, Madison, WI) and had free access to food and water before randomization by weight. Mice were administered either FGF19 or FGF21 for a period of 7 days via continuous infusion using osmotic minipumps (ALZET, Cupertino, CA) at the doses specified. Following sacrifice glucose levels were determined using Precision G Blood Glucose Testing System (Abbott Laboratories, Abbott Park, IL).

### FGF treatment of ob/ob animals

Male ob/ob mice (n = 6 per group) (Taconic Farms) were maintained on a standard chow diet (Purina, 5001) and had free access to food and water before randomization by weight. Mice were administered either FGF19 (1 mg/kg/day) or FGF21 (1 mg/kg/day) for a period of 7 days via continuous infusion using osmotic minipumps (ALZET, Cupertino, CA). Following sacrifice glucose levels were determined using Precision G Blood Glucose Testing System (Abbott Laboratories, Abbott Park, IL).

### Antagonism of FGF21 action by ΔN17 in ob/ob mice

Male ob/ob mice (n = 6 per group) (Harlan, IN) were maintained on a standard chow diet (Purina, 5001) and had free access to food and water before randomization by weight. Mice were administered with either FGF21, ΔN17 or a combination of both via daily injection at doses indicated for a period of 3 days after which serum was collected for analysis. Prior to sacrifice and blood collection on day 3 the fasted cohorts were deprived of food overnight. Following sacrifice glucose levels were determined using Precision G Blood Glucose Testing System (Abbott Laboratories, Abbott Park, IL).

### BRDU incorporation assay

On day 1 of the study, an osmotic minipump (ALZET, Cupertino, CA) containing 5-bromo-2-deoxyuridine (16 mg/ml; BrdU, Sigma Aldrich) was implanted subcutaneously into each 9-week-old male C57bl/6J mouse (n = 10 per group; Charles River Laboratories, Charles River, MA). Each mouse was given daily subcutaneous injections of either phosphate-buffered saline (PBS, vehicle), FGF19 (2 mg/kg/day) or FGF21 (2 mg/kg/day) for 7 consecutive days. At the end of the 7-day study samples of liver were collected from each mouse, placed in 10% neutral-buffered formalin, processed routinely, and embedded in paraffin. Multiple tissue sections were produced from each paraffin block, stained with Hematoxylin & Eosin (H&E), or immunolabeled for BRDU by routine immunohistochemical methods as outlined below. The H&E tissue sections were evaluated routinely for microscopic changes [15]. BRDU-immunolabeled sections were used to enumerate BRDU-positive nuclei per 200× microscopic field and for evaluating the pattern and distribution of BRDU-positive hepatocellular nuclei.

Cellular incorporation of BrdU was detected by digesting deparaffinized tissue sections with 0.1% protease (Sigma Aldrich) and treating the sections with 2N hydrochloric acid. Sections were blocked with CAS BLOCK (Zymed Laboratories Inc., San Francisco, CA), incubated with a rat antibody to BrdU (Accurate, Westbury, NY), and bound rat antibody was detected with biotinylated rabbit antibody to rat IgG (Vector Laboratories, Burlingame, CA; catalogue no. BA 4001, lot no. S0907). Tissue sections were quenched with Peroxidase Blocking Solution (DAKO Corp, Carpinteria, CA) and retained biotin was detected with Vectastain Elite ABC kit (Vector Laboratories). Reaction sites were visualized with DAB Substrate-Chromagen System (DAKO Corp, Carpinteria, CA) followed by DAB enhancer (Invitrogen, Carlsbad, CA). Sections were counterstained with hematoxylin.

### Surface plasmon resonance (BiaCore) studies

BiaCore studies were performed on a BiaCore 2000 instrument (BiaCore, Inc., Uppsala, Sweden). Proteins were covalently immobilized on censor chip CM4 using amine coupling according to the manufacturer's protocol. Typically, 100–50 response units (RU) were immobilized on individual flow cells of the sensor chip. BSA (Pierce Chemical; Rockford, IL) was immobilized on flow cell 1 as a negative control. Proteins suspended in HBS-P (BiaCore, Inc.) were then injected for 30 min at a flow rate of 30 ml/min using the kinject command. KD kinetic constants were calculated by BiaEvaluation 4.1 software using a 1∶1 Langmuir model.

### Glucose uptake assays

Cells were treated as indicated for 3 h and glucose uptake was assayed as previously described [16]. 3T3-L1 adipocytes were differentiated as previously described [16] and treated as indicated prior to assessment of glucose uptake.

### ERK1/2 phosphorylation assays

Cells were treated as indicated for 5 min and subsequently lysed. Total ERK phosphorylation was assessed using an AlphaScreen SureFire Phospho-ERK1/2 Assay Kit (Perkin Elmer) according to the manufacturer's instructions and an EnVision Multilabel Microplate Reader Model 2103 (Perkin Elmer) with the AlphaScreen HTS Turbo option was used for signal detection.

### RNA isolation, RT and real-time quantitative PCR

RNA was isolated from tissues using TRIzol reagent (Invitrogen, Carlsbad, CA) or by homogenization of frozen samples in Lysing Matrix D shaker tubes (MP Biomedicals, Santa Ana, CA) and was reverse transcribed into cDNA using a High-Capacity cDNA Reverse Transcription Kit (PE Applied Biosystems, Foster City, CA). Reactions were performed in triplicate on an ABI Prism 7900HT (PE Applied Biosystems) and were normalized to either 36B4 mRNA or 18S rRNA. ssays-on-Demand Gene Expression Products (PE Applied Biosystems) were as follows: hEGR1, Hs00152928_m1; hFGFR1, Hs00915142_m1; hFGFR2, Hs01552926_m1; hFGFR3, Hs00179829_m1; hFGFR4, Hs01106908_m1; hKL, Hs00183100_m1; hKLB, Hs00545621_m1; mFGFR1, Mm00438930_m1; mFGFR2, Mm01269930_m1; mFGFR3, Mm00433294_m1; mFGFR4, Mm01341852_m1; mKL, Mm00473122_m1; mKLB, Mm00502002_m1; rFGFR1, Rn00577234_m1, rFGFR2, Rn01269940_m1; rFGFR3, Rn00584799_m1; rFGFR4, Rn01441815_m1; rKL, Rn00580123_m1.

### Statistical analysis

Data are presented as mean ±SEM. Statistical analysis was performed using one-way ANOVA, followed by Dunnett's multiple comparisons test where appropriate. Differences were considered significant when P = <0.05.

## Results

**Figure 1:**
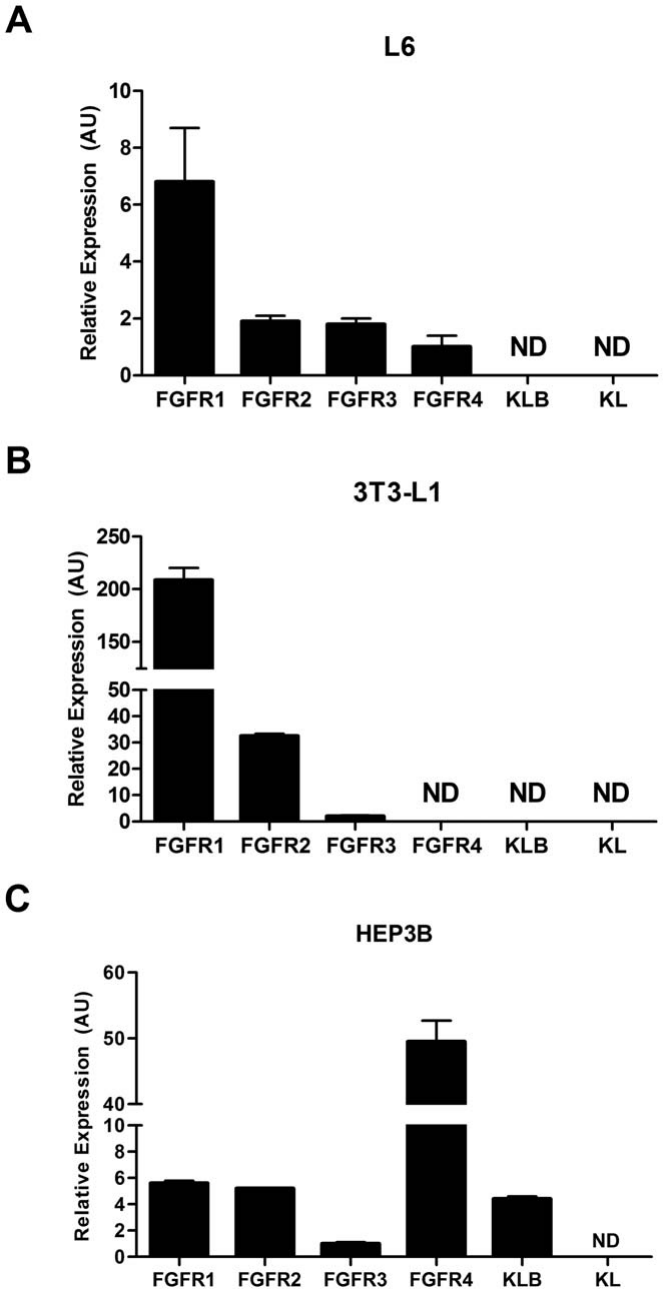
Expression of FGF receptors and Klotho co factors in cell culture models. In 3T3-L1 cells we found a high level of FGFR1 expression along with modest levels of FGFR2 and FGFR3. In these cells FGFR4, KL and KLB were not detectable (A). In Hep3B cells there was detectable expression of all 4 FGF receptor subtypes, however, we detected especially high levels of FGFR4. Hep3B cells were also found have appreciable expression of KLB while KL was not detectable (B). In L6 cells expression of all FGFRs was extremely low in comparison to other cells lines we screened in addition to undetectable levels of KLB at baseline (C).

Prior to testing FGF19 and FGF21 for activity in cell based assays we measured expression of FGF receptors and Klotho subtypes in the cell lines we used via RT-qPCR. We found that the expression of FGFR isoforms and the Klotho co-factors differed greatly between the lines. In 3T3-L1 fibroblasts we saw high levels of FGFR1 in addition to lower expression of FGFR2 and only traces of FGFR3 with no detectable FGFR4, KL or KLB (Figure 1A). In Hep3B cells there were very high amounts of FGFR4 with modest levels of FGFR1, FGFR2 and KLB, low FGFR3 and no detectable KL (Figure 1B). In L6 cells the expression of all the FGFRs was extremely low when compared to the other cell lines we analyzed, KL and KLB were not detectable (Figure 1C).

**Figure 2:**
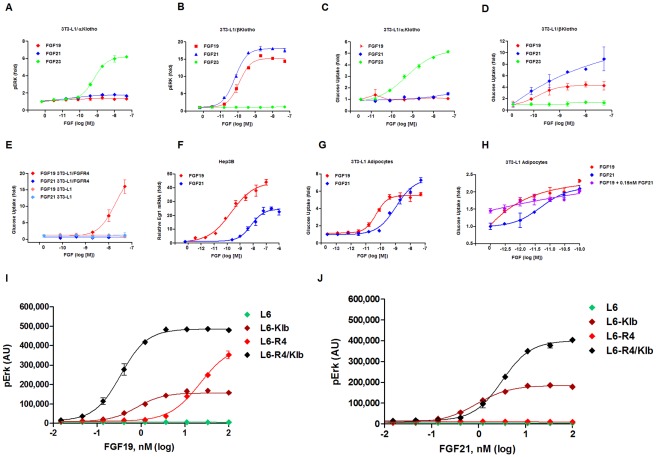
The “hormone like” FGFs exhibit different signaling properties in vitro. *Panel 1.* In 3T3-L1 fibroblasts over-expressing KL we saw phosphorylation of the FGF target ERK upon treatment with FGF23, while FGF19 and 21 had no effect (A). Conversely, in 3T3-L1/KLB cells we saw no effect of FGF23 but potent signaling with FGF19 or FGF21 treatment (B). When we examined glucose uptake in the 3T3-L1/KL cells we found that only FGF23 lead to its stimulation (C). As we saw with pERK in 3T3-L1/KLB cells FGF19 and FGF21 both increased glucose uptake significantly (D). In 3T3-L1 fibroblasts expressing FGFR4 in the absence of KLB only FGF19 was able to increase glucose uptake (E). Furthermore, in Hep3B cells which show a relative enrichment of FGFR4, FGF19 was significantly more potent than FGF21 in inducing expression of the immediate early gene EGR1 (F). When 3T3-L1 cells were differentiated to become mature adipocytes FGF19 was also more potent than FGF21 even in the absence of FGFR4 expression suggesting a possible unknown factor which is not present on fibroblasts may be affecting FGF19's action in these cells, or vice versa (G). In 3T3-L1 adipocytes treated with either FGF19, FGF21 or a combination of both we did not see any additive or synergistic effects of combination treatment over individual therapy suggesting FGF19 and FGF21 share a common mechanism of action (H). To confirm our initial results regarding the specificity of FGF19 for FGFR4 we turned to L6 cells which have been reported to have extremely low expression of FGFRs and KLs. In parental L6 cells treatment with FGF19 had no effect on the level of ERK phosphorylation, however, when cells were transfected with KLB a small but significant increase in pERK was detected. Furthermore, when FGFR4 was added the response to FGF19 stimulation was magnified. Interestingly, we saw again that in cells transfected with FGFR4 alone FGF19 was also able to induce pERK confirming KLB independent signaling with this ligand can occur (I). In contrast to FGF19, cells treated with FGF21 showed pERK induction only in the presence of KLB with the level of this baseline induction similar to that seen with FGF19 treatment (J).

In order to assess the specificity and functional significance of the interaction between FGF19, FGF21, FGF23 and the Klotho family we conducted studies in which we expressed either KL or KLB in 3T3-L1 fibroblasts. We chose 3T3-L1 fibroblasts as neither KL or KLB is natively present in these cells [6]. Firstly, we examined phosphorylation of ERK, an event known to be downstream of FGF receptor activation [17]. The addition of KL to 3T3-L1 cells to led to a robust induction of pERK following stimulation with FGF23. There was no effect of either FGF19 or FGF21 in 3T3-L1/KL cells (Figure 2A). However, when we treated KLB expressing cells we saw a very potent induction of pERK by FGF21 and to a slightly lesser degree FGF19, but no effect with FGF23. We then examined the capability of the FGFs to induce glucose uptake in the same cell lines to provide a functional readout of their activity. As in our pERK assay only FGF23 was able to significantly induce glucose uptake in the KL expressing cells (Figure 2C). In 3T3-L1/KLB cells FGF21 and FGF19 both induced glucose uptake with FGF21 again slightly more potent (Figure 2D) as we have reported previously [6]. Next we expressed FGFR4 in 3T3-L1 fibroblasts to confirm FGF19 activity in the absence of KLB. As predicted only FGF19 was able to significantly increase glucose uptake in 3T3-L1/FGFR4 cells (Figure 2E). To determine if receptor isoform distribution was mediating the difference in potency of the factors we assayed the ability of FGF19 and FGF21 to induce the immediate early gene early growth response 1 (EGR1) in Hep3B cells which express a high level of FGFR4. In these cells we found that FGF19 was very potent in terms of its ability to induce EGR1 gene expression. FGF21 did induce EGR1 in these cells but with reduced efficacy when compared to FGF19 (Figure 2F).

To determine if differentiation from fibroblast to adipocyte alters the response of 3T3-L1 cells to the hormone-like FGFs we treated 3T3-L1 adipocytes with either FGF19 or FGF21 and assessed their effects on ERK phosphorylation and glucose uptake. The expression of FGFRs in these cells is very similar to that which we showed for 3T3-L1 fibroblasts [18]. However, as we have previously reported the one major difference in terms of hormone-like FGF signaling capacity in these cells is that 3T3-L1 adipocytes unlike 3T3-L1 fibroblasts express KLB [6]. In these cells FGF19 showed increased potency over FGF21 which was evident in our glucose uptake assay (Figure 2G). Importantly, when 3T3-L1 adipocytes were treated with either FGF19, FGF21 or a combination of both we did not observe any synergistic or additive effects suggesting an identical receptor activation mechanism (Figure 2H).

As both 3T3-L1 and Hep3B cells express appreciable amounts of FGFRs we turned to L6 cells to confirm our findings as these cells have been reported previously to have vanishingly low expression of both FGFRs and KLs [19]. In parental L6 cells there was no detectable induction of ERK phosphorylation with either FGF19 or FGF21 treatment (Figure 2I & J). Contrary to the notion that L6 cells do not possess sufficient FGFR expression to permit signaling, we saw a similar but appreciable induction of pERK in both FGF19 and FGF21 treated cells. Addition of FGFR4 in the presence of KLB caused a significant increase in pERK in the FGF19 treated cells (Figure 2I). Treatment of FGFR4/KLB cells with FGF21 also led to a significant increase in pERK, however, in comparison to FGF19 the effect was significantly less potent (Figure 2J). Supporting our earlier data in 3T3-L1/FGFR4 cells we also saw an induction following FGF19 stimulation in cells expressing FGFR4 in the absence of KLB (Figure 2I). In the same cells there was no change in pERK following FGF21 treatment further reinforcing KLB independent R4 mediated pERK induction as a defining feature separating the two factors (Figure 2J). These findings align well with the fact that only FGF19 is able to directly bind FGFR, and only one variant, FGFR4, with 6.5 nM affinity in a KL and-KLB-independent manner. This was evident from our binding analysis of FGF19, FGF21, and FGF23 with various FGFR-Fc constructs (data not shown).

**Figure 3:**
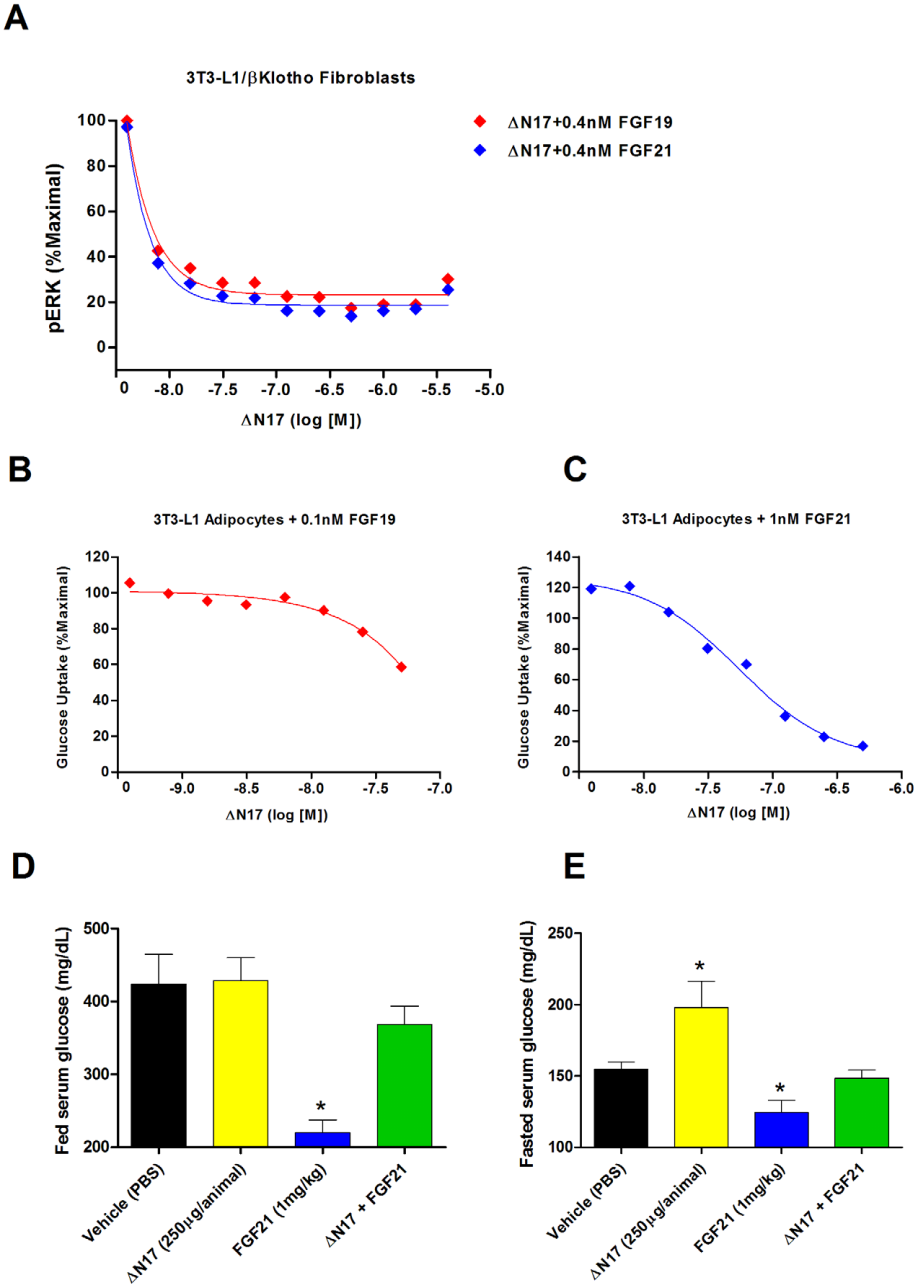
Inhibition of FGF21 signaling also blocks FGF19 action in vitro. *Panel 1.* In 3T3-L1/KLB fibroblasts co-treatment with the competitive agonist ΔN17 was able to block the induction of ERK phosphorylation caused not only by FGF21 but also by FGF19 (A). In our glucose uptake assay in 3T3-L1 adipocytes ΔN17 also suppressed the activity of both FGF19 (B) and FGF21 (C). *Panel 2.* To determine if the inhibition of FGF19/21 signaling we see in vitro translates to effects on metabolic parameters in vivo we examined fed and fasted glucose levels in ob/ob mice treated with FGF21, ΔN17 or a combination of both. In fed mice treatment with ΔN17 alone had no effect on serum glucose. FGF21 treatment reduced glucose levels significantly in the fed state, however, when the two treatments are combined the effect of FGF21 to reduce glucose is abolished (D). In fasted animals FGF21 again reduced glucose, an effect blocked by combination with ΔN17. Interestingly, we found that in fasted animals treatment with ΔN17 partially blocked the normal reduction in the serum glucose, suggesting ΔN17 may interfere in the regulation of glucose homeostasis (E).

We have previously demonstrated that N terminally truncated FGF21 (ΔN17) acts *in vitro* as a competitive antagonist and leads to inhibition of FGF21 mediated signaling by binding to KLB and blocking FGF21 mediated receptor activation [13]. As FGF19 in the context of KLB expression is able to activate FGFRs other than FGFR4 ([6] Figure 2D) we sought to test if ΔN17 could also inhibit the action of FGF19. Indeed, in 3T3-L1 fibroblasts expressing KLB treatment with ΔN17 was able to block the induction of ERK phosphorylation caused not only by FGF21 but also by FGF19 (Figure 3A). Furthermore, in our glucose uptake assay in 3T3-L1 adipocytes ΔN17 also suppressed the activity of both FGF19 and FGF21 (Figure 3B & 3C).

To determine if the inhibition of FGF19/21 signaling we see in vitro translates to effects on metabolic parameters in vivo we examined fed and fasted glucose levels in ob/ob mice treated with FGF21, ΔN17 or a combination of both. In fasted animals FGF21 reduced glucose, an effect blocked by combination with ΔN17. Interestingly, we found that in fasted animals, treatment with ΔN17 partially blocked the reduction in serum glucose one normally observes in fasted animals, suggesting ΔN17 may interfere endogenous glucose homeostasis in the fasted state (Figure 3E). Furthermore, these data support a critical role for FGF19/FGF21 signaling in regulating serum glucose levels in the fed to fasted transition.

**Figure 4:**
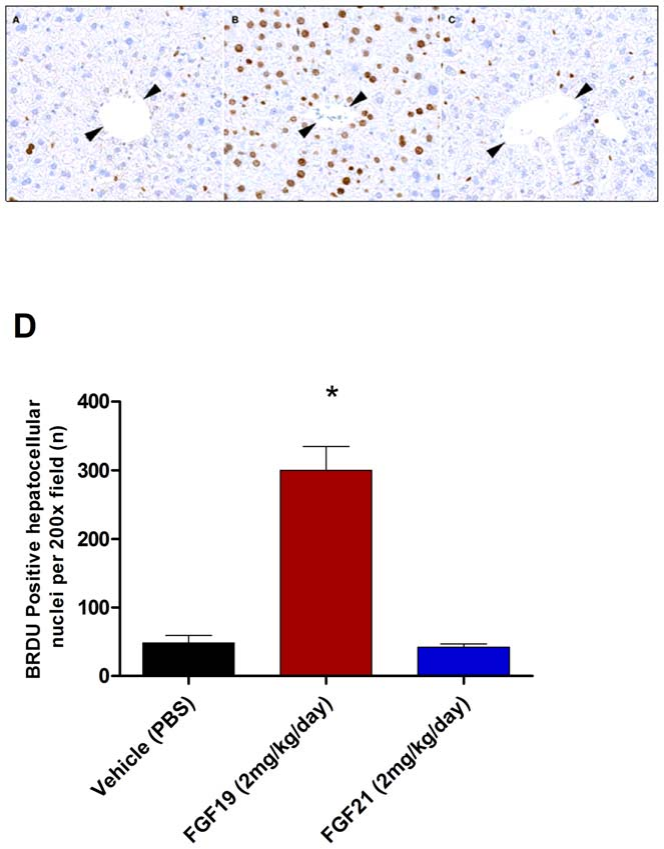
FGF19 treatment leads to hepatocellular proliferation. All mice received a constant infusion of BRDU during the 7 days of treatment. At the end of treatment samples of liver were harvested, preserved in formalin, processed routinely, and embedded in paraffin. Tissue sections were cut, immunolabeled for BRDU, and counterstained with hematoxylin. Representative sections are shown here from mice receiving injections of phosphate buffered saline (PBS; A), FGF19 (B), or FGF21 (C). The average number of BRDU-positive nuclei per 200× microscopic field is shown. Mice administered FGF19 had a statistically significant increase (* = p<0.0001) in the numbers of hepatocytes with BRDU-positive nuclei when compared to mice administered PBS (D). In contrast to FGF19, FGF21 did not induce hepatocellular proliferation. The arrowheads indicate the location of the centrilobular veins.

FGF19 has previously been reported to induce mitogenicity in animals [18], [20], [21]. Therefore, we assessed the effects of both FGF19 and FGF21 on hepatocellular proliferation in an in vivo model utilizing BRDU incorporation. FGF19 treated mice had a significantly greater number of BRDU positive hepatocellular nuclei when compared to vehicle treated animals (Figure 4A-C). To date there have been no reports of cellular proliferation associated with FGF21 treatment [14]. Consistent with this, there was no significant difference in the number of BRDU positive nuclei when compared to the vehicle group (Figure 4D).

**Figure 5:**
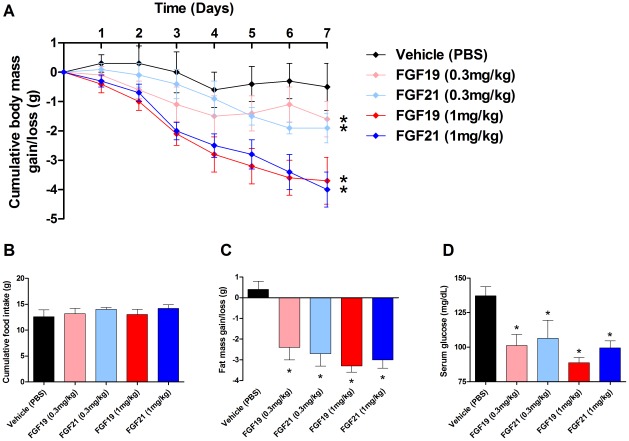
Treatment of DIO mice with either FGF19 or FGF21 improves metabolic dysfunction. Administration of recombinant FGF19 or FGF21 led to a reduction in body mass in a dose dependent fashion (A). Both FGF19 and FGF21 therapy led to a trend towards increased food intake, however, these differences were not significant (B). Treatment with FGF19 or FGF21 significantly reduced adiposity at both doses tested again in a dose dependant manner (C), All FGF treatments caused a significant reduction in serum glucose in DIO animals, furthermore, the reduction in glucose observed with the two proteins was strikingly similar (D).

Our group and others have previously reported on the efficacy of FGF21 in the treatment of obesity in animal models [22]. To compare the metabolic effects of FGF19 treatment to those seen with FGF21 we examined the metabolic effects of chronic administration of the factors to high fat diet induced obese c57BL/6 mice. FGF19 therapy reduced body mass in a dose dependent fashion (Figure 5A) with approximately 3.7 g difference between the highest dose of FGF19 (1 mg/kg) and the vehicle treated animals. FGF21 treatment also led to a dose dependant reduction in body mass with a 4 g reduction in weight in the 1 mg/kg treated animals. This weight loss occurred in the absence of any significant effects on caloric intake with either FGF19 or FGF21 (Figure 5B). MRI measurements of body composition showed the weight loss was primarily due to reduced fat mass in both FGF19 and FGF21 treated cohorts (Figure 5C). Furthermore, all FGF19 and FGF21 treated animals exhibited a significant drop in serum glucose which did not show dose dependency although there was a trend toward increased efficacy at the 1 mg/kg dose in the FGF19 treated cohorts when compared to FGF21 (Figure 5D).

**Figure 6:**
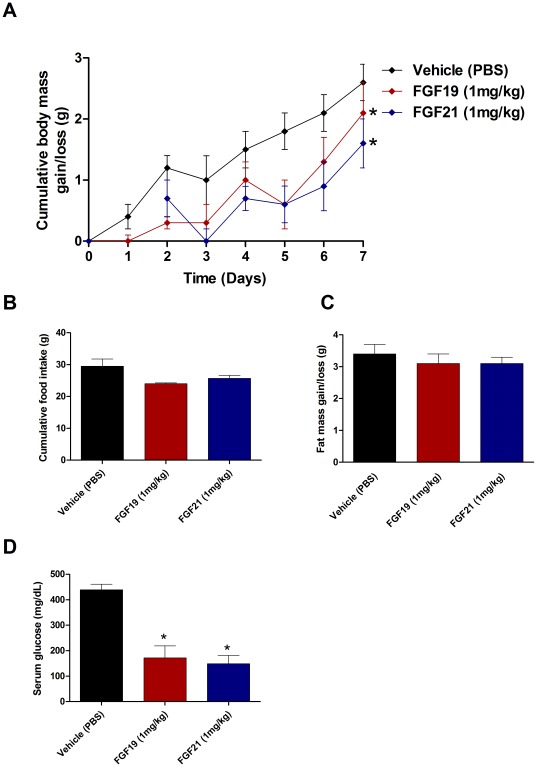
Treatment of ob/ob mice with either FGF19 or FGF21 improves metabolic dysfunction. In ob/ob mice neither FGF19 nor FGF21 were able to reduce body mass significantly; however, both treatment groups exhibited significant reductions in body mass accrual over the 7 day treatment period (A). Food intake was significantly reduced in the FGF19 treated mice while FGF21 treatment caused a trend to reduced caloric intake (B). No difference in adipose mass was observer following administration of either FGF19 or FGF21 (C) While the ob/ob mice showed significantly less profound effects on body mass and adiposity than was observed in the DIO group the glucose lowering following treatment with either FGF19 or FGF21 was still very significant suggesting possible partitioning of the effects of the endocrine FGFs (D).

In an effort to compare efficacy of the factors in another model of obesity we examined the effects of chronic FGF19 and FGF21 infusion in ob/ob mice. Interestingly ob/ob mice seem to display a differential response to FGF treatment when compared to WT mice. In both the FGF19 and FGF21 treatment groups there was a significant attenuation of body mass accrual over the 7 day treatment period, furthermore, the magnitude of the effect was greater in FGF21 treated animals when compared to FGF19 treatment (Figure 6A). FGF19 treatment led to a significant reduction in food intake, while there was also a trend to reduced food intake in the FGF21 treated animals it did reach statistical significance (Figure 6B). In spite of lower body mass in the FGF treated animals there was no significant difference in fat mass in either group (Figure 6C). Consistent with earlier reports administration of either FGF19 and FGF21 led to significant reductions in serum glucose in ob/ob mice, however, the magnitude of the effect was equivalent for both factors (Figure 6D).

## Discussion

The therapeutic potential of both FGF19 and FGF21 in the treatment of metabolic disorders has been discussed extensively in the literature [7], [9], [14], [20]. Several studies have now demonstrated that administration of FGF19 or FGF21 can have beneficial effects in rodent and primate models of obesity and diabetes [10], [20], [23], [24]. However, despite the similarities in the actions of the two factors there has been no direct comparison of their effects to date.

Our group and others have previously reported that the metabolic activity of FGF21 or lack of thereof is determined by the presence or absence of the cofactor KLB [6], [17], [25]. Here we show in 3T3-L1 fibroblasts that the over-expression of either KL or KLB permits signaling by specific FGFs. When cells express KL we observe that FGF23 treatment leads to both increased phosphorylation of ERK and as well as manifestation of functional effects such as increased glucose uptake. In 3T3-L1/KL cells we did not see any effect of FGF19 or FGF21 on either signaling or glucose uptake confirming specificity for FGF23. This is an important observation as it has been reported previously that FGF19 was able to bind KL and induce signaling in KL-expressing cells [26].

In 3T3-L1/KLB fibroblasts we saw FGF19 and FGF21 mediated signaling and glucose uptake with FGF21 more potent than FGF19. We did not see any effect of FGF23 in the 3T3-L1/KLB cells consistent with previous data showing specificity for KL alone [27]. In a somewhat surprising result we found that if 3T3-L1 fibroblasts were differentiated to become adipocytes, FGF19 becomes more potent than FGF21 in inducing both pERK and glucose uptake. Interestingly, the sensitivity of 3T3-L1 adipocytes to FGF19 is higher than that observed with FGF19 treatment in the 3T3-L1/KLB fibroblasts suggesting an as yet unknown factor which modulates FGF19 action may be present in adipoctyes but absent in fibroblasts, or vice versa. Given the fact that FGF21 activity is similar on both 3T3-L1 adipocytes and 3T3-L1/KLB fibroblasts this factor is likely FGF19 specific. Furthermore, we show that FGF19 is able to act on adipocytes with higher potency than FGF21. This is a novel observation as FGF19 was previously considered to act predominantly on cells of hepatic origins and liver [28].

In cells which predominantly express FGFR4 but do not express KLB, FGF19 was active but FGF21 was not. In the presence of KLB, both FGF19 and FGF21 can signal via FGFR4 but FGF19 appears to be significantly more potent than FGF21. This was evident in 3T3-L1 fibroblasts stably expressing FGFR4 in which FGF19 was able to act directly in the absence of KLB and induce glucose uptake, while FGF21 did not have any activity. This finding is important as while previous reports have shown binding of FGF19 to FGFR4 [18], [20], [21], our report is the first to show that the physical FGF19/FGFR4 interaction leads to subsequent activation of FGFR4. Our data also contrasts with previously the suggested hypothesis that FGF21 cannot signal via FGFR4 and act directly on cells of hepatic origin [29], [30], but is in agreement with a recent report demonstrating FGF21 signaling in the liver [17]. In our experiments FGF21 was significantly less potent than FGF19 in liver-derived Hep3B cells which do express detectable levels of KLB, however, it clearly is able to signal in these cells (Figure 2F). The differences in FGF19 and FGF21 potencies could be due to the presence of high levels of FGFR4 in these cells and differential ability of FGF19 and FGF21 to activate this FGFR. To test this association we investigated FGF19 and FGF21 action in L6 myoblasts which have previously been employed extensively in FGF signaling assays due to extremely low endogenous expression of FGFRs and KLs [19]. Consistent with our earlier result in 3T3-L1/FGFR4 cells (see Figure 2E) we also found in L6 cells that FGF19 but FGF21 not is able to signal in via R4 in the absence of KLB. Nevertheless, FGF19 action was significantly improved when KLB was co-expressed with FGFR4 (Figure 2I), and importantly we show that again FGF21 is able to activate FGFR4 in the context of KLB co-expression.

On a side note, while L6 cells have previously been reported to be free of background signaling due to a paucity of FGFR expression [19], [31] we see an appreciable background signal in cells transfected with KLB alone with both FGF19 and FGF21 treatment. As FGF19 and FGF21 signaling in these cells can be blocked with FGFR inhibitor (data not shown) these data suggest sufficient FGFR 1–3 expression levels in L6 cells to allow detectable signaling. It is unlikely that the FGFR permitting this signal is FGFR4 as FGF19 cannot signal in the parental cell line without FGFR4 over expression.

We found no synergistic or additive effect on glucose uptake when cells were treated with both FGF19 and FGF21 simultaneously. This indicates that these two factors share a common signaling pathway via which they regulate glucose transport in cell culture.

This commonality of the two factors extended to our studies of inhibition using the competitive antagonist ΔN17 which we have previously shown is effective in inhibiting FGF21 at the receptor activation level in vitro. In the present study we observed that not only does ΔN17 inhibit downstream FGF21 signaling but also shows a similar efficacy in blocking FGF19 mediated effects. These data support the hypothesis that in cell culture models FGF21 and FGF19 operate by activation of a similar signaling cascade. Furthermore, we go on to demonstrate that *in vivo* ΔN17 is also able to block the glucose lowering action of exogenous FGF21 in both fed and fasted mice. In both fed and fasted ob/ob mice treated with FGF21 we see the usual glucose lowering effect we have reported previously [10]. However, when FGF21 was co-administered with ΔN17 FGF21s glycemic effects were completely abolished (see Figure 3D). As ΔN17 acts as a competitive agonist to prevent FGF21 and FGF19 interaction with KLB and subsequent FGFR activation, this result establishes the critical role of KLB to propagate glucose lowering action of FGF19/FGF21 in vivo. This is a very novel and critical finding since to date KLBs co-receptor function for FGF19/FGF21 has been shown only in vitro [6], [13], [32] and uncertainty exists as to whether KLB is required for FGF21 action in vivo [28].

It is also important to note that *in vivo* administration of dN17 alone affected plasma glucose but only in the fasted state. Given the KLB antagonistic nature of ΔN17s mode of action, and the absence of effects on glucose homeostasis in a fed mice treated with the protein, we hypothesize that even though a substantial amount of FGF21 is detected in plasma of fed ob/ob mice, it is likely present in a non-functional form which is unable to interact with endogenous KLB in the manner described previously [22], [33]. In contrast, significantly increased levels of FGF21 plasma levels during fed to fast transition have been reported previously in animals [34], [35], and we confirmed this data in ob/ob mice (data not shown). Thus, as ΔN17 is active on its own only in food-deprived mice, fasting is likely a condition at which FGF21 is present in mouse blood in its active, KLB interacting form. This observation is novel and may call into question recent publications debating the presence or absence of FGF21 resistance in obese states [36], [37].

As several previous studies have noted mitogenic effects in animal models following treatment with FGF19 and absence of thereof with FGF21, we examined both FGF19 and FGF21 in an in vivo setting. In our hands FGF19 dosing led to a very significant increase in proliferation in the liver while FGF21 had no effect. Our data support earlier work suggesting FGFR4 binding by FGF19 may mediate its mitogenic effects [38] and that blockade of FGFR4 may be beneficial to treat proliferative diseases [39]. These results, taken alongside the in vitro signaling differences between FGF21 and FGF19 suggest that FGFR4 engagement and/or the level of its activation may lead to functionally different effects than those seen with activation of other FGFRs. Studies using truncated forms of FGF19 have shown that activation of FGFR4 is essential for the proliferative effect seen with FGF19 treatment [18]. These data suggest that modification of FGF19 to eliminate FGFR4 interaction while retaining binding to other FGFRs may yet provide a possible avenue for potential therapies [18].

Supporting this hypothesis is the finding that treatment with FGF19 improves glucose tolerance in DIO FGFR4KO mice suggesting activation of FGFR4 is not required in the mediation of at least some of the metabolic effects of this factor [40]. Both our data and studies in the literature show that FGF19 and FGF21 can bind and activate multiple FGFRs in the presence of KLB [11], [17], [41], and contrasts with the previous notion that FGF19 activity is strictly liver and FGFR4-specific [29]. Therefore it is likely that the FGFR1/KLB complex possibly with contributions from FGFR2 and FGFR3 are primary mediators of the positive metabolic effects of FGF19 and FGF21. Nevertheless, the phenotype of FGFR4 knock-out animals is also suggestive of a metabolic role of this receptor [12].

To date direct comparisons of FGF19 and FGF21 treatment in animal models have not been conducted. Here we show in DIO mice both FGF19 and FGF21 have beneficial effects in the treatment of metabolic dysregulation. It has been previously demonstrated that in DIO models ranging from rodents to primates that FGF21 treatment is able to correct the abnormal metabolic parameters evoked by prolonged high fat diet feeding [22]. In genetic models of obesity such as the ob/ob mouse either direct treatment with FGF21 or its induction via feeding of a high fat very low carbohydrate diet leads to weight loss and metabolic improvement [10], [42]. Our current data support these previous publications and show that in DIO mice FGF21 treatment is extremely effective in correcting metabolic dysfunction. Studies on the metabolic effects of FGF19 have been much more limited in scope, likely due to the known mitogenic effects of FGF19. FGF19 therapy has been shown to be effective in treatment of the metabolic disturbances observed in DIO and ob/ob mice [11], [20]. These data are supported by studies demonstrating that over-expression of FGF19 on the ob/ob background leads to a significant amelioration of the ob/ob phenotype [24].

Here we show that the metabolic effects of treatment with either FGF19 or FGF21 are almost identical. The main difference noted was increased potency of FGF21 when compared to FGF19 in terms of its effect on weight loss. Other effects such as the glucose lowering component of their action were indistinguishable, supporting the hypothesis of a shared mechanism of action.

In conclusion, our study demonstrates that the effects of FGF19 and FGF21 both in vitro and in vivo show a high degree of similarity. This interchangeability between the factors likely results from the ability of both to bind KLB and FGFRs. In mice, treatment with FGF19 and FGF21 both led to amelioration of the obese phenotype with significant improvements in all parameters tested. Our data demonstrate that both in vitro and in vivo FGF19 and FGF21 are able to potently activate the KLB/FGFR complex and that this activation likely mediates the positive metabolic outcomes we observe. Our data lend further support for further investigation of both FGF21 and FGF19 as potential therapies for obese/diabetic humans.
